# Supplementary material for: Identification of photoperiod-induced specific miRNAs in the adrenal glands of Sunite sheep (Ovis aries)
Source: Front Vet Sci. 2022 Jul 22;9:888207. doi: 10.3389/fvets.2022.888207 (PMC9354845; doi:10.3389/fvets.2022.888207)
Supplement: Supplementary file 1 [file Data_Sheet_1.ZIP › Supplementary file/miRDeep2 file.zip!/miRDeep2 file/Novel_Structure/SPLP42A2/Novel_340.pdf]

Provisional ID	:	NC_019460.2_8517
Score total	:	2.3
Score for star read(s)	:	-1.3
Score for read counts	:	0
Score for mfe	:	2
Score for randfold	:	1.6
Score for cons. seed	:	
Total read count	:	255
Mature read count	:	255
Loop read count	:	0
Star read count	:	0

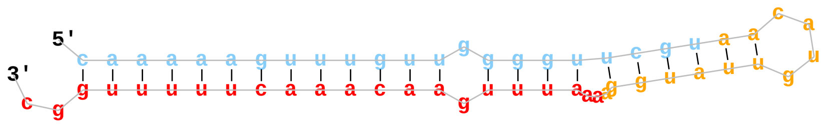

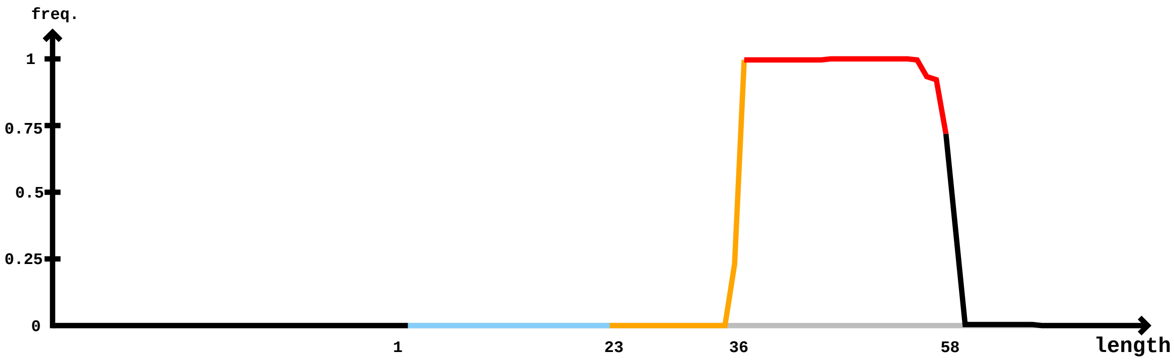

Star Mature

```
5'- accacacucuggaaaucaguaaauauuggguugguCaaaaaguuuguugggguucguaacauguuauggaaaauuugaacaaacuuuuuggccaacccaguauuauaaacca   -3'    exp
    .(((.....)))...................((((((((((((((((((((((((((.((((((((((....))))))...)))).))))))))))))))))))))))))))........    reads      mm         sample
    .....................................................................aaaauCugaacaaacuuuu........................    1          1          seq
    .....................................................................aaaauCugaacaaacuuuuu.......................    15         1          seq
    .....................................................................aaaauCugaacaaacuuuuugg.....................    39         1          seq
    .....................................................................aaaauCugaacaaacuuuuuggc....................    4          1          seq
    ......................................................................aaauCugaacaaacuuuuu.......................    1          1          seq
    ......................................................................aaauCugaacaaacuuuuug......................    3          1          seq
    ......................................................................aaauCugaacaaacuuuuugg.....................    13         1          seq
    ......................................................................aaauuugaacaaacuuuuuggc....................    1          0          seq
    ......................................................................aaauCugaacaaacuuuuuggc....................    177        1          seq
    ......................................................................aaaCuugaacaaacuuuuuggc....................    1          1          seq
    ...............................................................................caaacuuuuuggccaacccaUu...........    1          1          seq
```
